# Railway Spine

**Published:** 1894-10

**Authors:** 


"RAILWAY SPINE."—As we predicted, the paper of Dr. Swearingen, in our July number, has "brought on a general engagement," and firing all along the line has commenced. In fact several *big guns* have began to boom, and the rattle of small arms is still to be heard (this, for instance, is one of them). Well, we have had our say, and we stand pat on it. The chip is still on our shoulder. Dr. Swearingen hit hard, and has received some hard hits in return, all of which, so far, and so far as we have heard, has been taken good naturedly. Dr. Wallace, in *Texas Sanitarian*, hits him some well-directed hits, but the hardest hit he hit him was a clear miss, as the Irishman would say: for instance, he says, Dr. S. is prejudiced against railroads, and it is from associations with the "ins." of the present administration. That was a misfit, and is clearly a begging of the question. A careful re-reading of the paper on our part, fails to reveal any language that evinces "prejudice."

Dr. Wallace challenges any one to cite an instance wherein any just claim against a railroad has not been paid in full. The JOURNAL can cite him to at least one—a bill of $30 for surgical services, in payment of which *ten dollars* was offered by the Chief Surgeon, with the alternative, "this or nothing." No doubt other physicians than the one here referred to, could give instances in which their fee has been fixed by the Chief Surgeon, irrespective of their claims.

